# Progress in the application of hydrogels in immunotherapy of gastrointestinal tumors

**DOI:** 10.1080/10717544.2022.2161670

**Published:** 2023-01-01

**Authors:** Hao Zheng, Meng Li, Lili Wu, Wenshang Liu, Yu Liu, Jie Gao, Zhengmao Lu

**Affiliations:** a Department of General Surgery, Shanghai Changhai Hospital, Naval Medical University, Shanghai, China; b Department of Dermatology, Shanghai Ninth People’s Hospital, Shanghai Jiaotong University, Shanghai, China; c Changhai Clinical Research Unit, Shanghai Changhai Hospital, Naval Medical University, Shanghai, China; d Department of Gastroenterology, Jinling Hospital, Medical School of Nanjing University, Jiangsu, China

**Keywords:** Hydrogel, drug delivery system, immunotherapy, tumor vaccine, gastrointestinal tumors

## Abstract

Gastrointestinal tumors are the most common cancers with the highest morbidity and mortality worldwide. Surgery accompanied by chemotherapy, radiotherapy and targeted therapy remains the first option for gastrointestinal tumors. However, poor specificity for tumor cells of these postoperative treatments often leads to severe side effects and poor prognosis. Tumor immunotherapy, including checkpoint blockade and tumor vaccines, has developed rapidly in recent years, showing good curative effects and minimal side effects in the treatment of gastrointestinal tumors. National Comprehensive Cancer Network guidelines recommend tumor immunotherapy as part of the treatment of gastrointestinal tumors. However, the heterogeneity of tumor cells, complicacy of the tumor microenvironment and poor tumor immunogenicity hamper the effectiveness of tumor immunotherapy. Hydrogels, defined as three-dimensional, hydrophilic, and water-insoluble polymeric networks, could significantly improve the overall response rate of immunotherapy due to their superior drug loading efficacy, controlled release and drug codelivery ability. In this article, we briefly describe the research progress made in recent years on hydrogel delivery systems in immunotherapy for gastrointestinal tumors and discuss the potential future application prospects and challenges to provide a reference for the clinical application of hydrogels in tumor immunotherapy.

## 1. Introduction

Gastrointestinal tumors are common malignant tumors that cause a huge medical burden and economic spending worldwide (Wang et al., 2021). According to Global Cancer Statistics in 2020, there were 19.3 million new cancer cases and 10 million cancer-related deaths reported worldwide, among which the incidence of colorectal cancer ranks third (10%), the death toll ranks second (9.4%), the incidence of gastric cancer ranks fifth (5.6%) and the death toll ranks fourth (7.7%) (Sung et al., 2021). In most cases, surgery is the first option for gastrointestinal tumors, and patients need systemic chemotherapy or radiotherapy after surgery to remove the tumor cells that may remain in their bodies (Chen et al., 2021). However, these postoperative treatments lack specificity for tumor cells, which leads to significant side effects and poor compliance of patients (Tempero et al., 2021). Tumor immunotherapy has always been an important part of comprehensive treatment (Wang et al., 2021). Wiliam Coley, who has since become the father of tumor immunotherapy, successfully reduced the tumor volume by injecting Streptococcus pyogenes into tumor patients in 1891 (Coley, 1991). Tumor immunotherapy is a therapeutic approach to kill and remove neoplasms by activating the body’s own immune system and using the immune active substances and immune cells produced by the body. Tumor immunotherapy is superior to traditional treatments such as surgery, radiotherapy and chemotherapy since tumor immunotherapy can not only eliminate primary tumors but also prevent neoplasm recurrence and eliminate local metastatic tumors by maintaining standing immune memory (Topalian et al., 2011). During the process of neoplasm immunotherapy, the body’s own immune system is activated, and then the body’s own immune cells and substances are activated to eliminate the tumor. Generally, tumor immunotherapy consists of four categories, including immune checkpoint blockade, tumor vaccines, adoptive T-cell immunotherapy, and oncolytic viruses (Khalil et al., 2016). Notably, immunotherapy has been included in the recommended scheme of gastrointestinal cancer treatment by the NCCN guidelines due to its good curative effect and low side effects in the therapy of gastrointestinal neoplasms.

The field of immunotherapy has achieved impressive advances; however, there are challenges and obstacles, such as limited response rates, inability to predict clinical effectiveness, and other factors, and potential side effects, including autoimmune reactions or cytokine release syndromes, insufficient activation of the immune system, fast drug release, and insufficient application of multiple drugs, remain and hamper the further adhibition of immunotherapies in clinics (Hegde & Chen, 2020). Furthermore, since neoplasm cells themselves can also release immunosuppressive substances, the immune response around the tumor is usually selectively suppressed (Baumeister et al., 2016; Gonzalez et al., 2018), which explains why many patients fail to respond to immunotherapy: it is an inability to activate the immune response around the neoplasm tissue rather than a failure to activate a systematic immune response (Baumeister et al., 2016; Pandya et al., 2016; Gonzalez et al., 2018; Sanmamed & Chen, 2018). Furthermore, tumor microenvironments contain a number of nonredundant factors that accelerate immunosuppression and inhibit anticancer responses. Physical barriers to immune infiltration are among them, as are the recruitment of suppressive immune cells and upregulation of molecules on tumor cells that bind to inhibitory receptors on immune cells. Attempts have been made to find ways to improve antitumor immunity and the immune system’s ability to suppress tumors to overcome this problem.

Hydrogels, which provide an ideal physiological environment for cargoes to be trans supported by interaction forces of different sizes, have become the first choice in recent years in many drug delivery systems and tissue engineering materials (Zhu et al., 2019). Chao et al. (2020) summarized the current situation and development trend of tumor immunotherapy based on injectable hydrogels and showed that it is a promising area that can inhibit systemic tumor growth and prevent side effects through local administration. Moreover, hydrogels have also been used as local depots for the delivery of single or multiple immunotherapy cargoes, such as traditional immunomodulatory factors, immune checkpoint blocking antibodies, and exogenous immune cells, through sustained release. When immunotherapy is combined with other therapies, such as chemotherapy, radiotherapy, and phototherapy, it can be even more effective for fighting tumors. Their use is widespread in many areas of biomedicine, especially in tumor treatment (Chen et al., 2019; Liu et al., 2020; Li et al., 2021).

In summary, hydrogels, as drug delivery systems, possess unique advantages in the immunotherapy of gastrointestinal tumors and offer great potential for the future development of new drugs. In this article, we review the development of hydrogel-based delivery systems and their use in immunotherapy of gastrointestinal tumors in recent years (Muraoka et al., 2019; Wang et al., 2020; Wu et al., 2021; Yang et al., 2021a, 2021b; Zhang et al., 2021; Zhu et al., 2021) (Table 1) and finally look forward to the future application prospects and challenges of hydrogel systems.

**Table 1.** Summary of the progress of hydrogels in immunotherapy of gastrointestinal cancer.

Types of immunotherapy	Hydrogel system	Embedded drug	Model of tumor cells	Result	Reference
Immune checkpoint inhibitor (anti-PD-L1)	P(ethylene-glycol)-Block-poly (L-leucine) Copolymers	REG and BMS202	CT-26	In neoplasm tissues, REG increased the expression of PD-L1. As a result of BMS202, more CD8+ T cells were infiltrated into tumors, more IFN-* and TNF-* were released	(Zhang et al., 2021)
Immune checkpoint inhibitor (anti-PD-L1)	Hydrogel of TMC	Polypeptide of OPBP-1	CT-26	As a result of OPBP-1 activation, CD8+ T cells are able to secrete more IFN-γ(interferon-γ) in human PBMCs	(Zhu et al., 2021)
Immune checkpoint inhibitor (anti-CTLA-4)	CHP: LPA + TLR9 +CPG OND	CTLA-4 Antibody	CT-26	As a result of competing with CD28 for B7 molecules on APC (CD80 and CD86), CTLA-4 could inhibit immature cells’ and TM cells’ early activation	(Muraoka et al., 2019)
Immunocyte	Hydrogel coloaded with polyphyllin II (PP2) and resiquimod(R848) (PR-Gel)	PP2 and R848	MFC	An increase in antitumor immunity and suppression of tumor growth were possible owing to PR-Gel’s ability to repolarize M2-like macrophages into M1-like macrophages	(Yang et al., 2021)
Tumor vaccine	PLEL hydrogels loaded with CpG and neoplasm lysates	CTX	CT-26	It was proven to suppress tumor growth and cause an immune response through cytotoxic T lymphocytes (CTLs)	(Yang et al., 2021)
Immunocyte	CDA-NT	CDA/CPT	CT-26	Immune-stimulating TME can be improved by CDA-NT, enhancing antitumor immunity	(Wang et al., 2020)
Immunocyte	Mel/G/DH + NIR (808 nm)	Melanin	CT-26	DCs can be activated in tumor-draining lymph nodes, enhancing CTL generation, and preventing tumor growth in a systemic immune answer	(Wu et al., 2021)

## 2. Classification and characteristics of hydrogels

It is possible to categorize hydrogels according to a number of characteristics, such as their origin, crosslinking nature, and degradation performance (Ishihara et al., 2002; Li et al., 2012; Spiller et al., 2012; Hussain et al., 2013; Lu et al., 2018; Czarnecka and Nowaczyk, 2020; Jiang et al., 2020) (Table 2). For example, hydrogels can be divided into two categories based on the origin of monomers: synthetic and natural materials (Cui et al., 2021). The most commonly used synthetic monomers are 2-hydroxyethyl methacrylate (pHEMA), polyvinyl alcohol (PVA) and polyethylene glycol (PEG), whereas the commonly used natural polysaccharide monomers are hyaluronic acid, alginate, gelatin, collagen, and DNA. Hydrogels made from synthetic materials can be produced easily industrially, modified chemically and have properties that can be precisely controlled and precise, but compared with natural polymer hydrogels, their biosafety, bioactivity and biodegradability are poor. Hydrogels can also be divided into two categories based on the crosslinking method: chemical crosslinking and physical crosslinking hydrogels. Chemical hydrogels are generated by chemical crosslinking between molecules, and the polymer chains are crosslinked in the form of covalent bonds to form a three-dimensional network structure. The cross-linking process is irreversible. The mechanical properties and stability of chemical hydrogels are their main advantages. The main component of a physical hydrogel is its three-dimensional network formed by noncovalent bonding between linear molecules, and the physical crosslinking points are formed by electrostatic, hydrogen bond, chain winding and hydrophobic forces between molecules. These hydrogels are generally reversible, and there is a moderate amount of preparation involved. According to different degradation properties, hydrogels can be classified as biodegradable or nonbiodegradable. Nonbiodegradable hydrogels refer to hydrogels that are insensitive to environmental factors and can keep their structure and physicochemical properties stable for a long time. Most natural hydrogels are biodegradable hydrogels, while most synthetic hydrogels are nonbiodegradable hydrogels.

**Table 2.** Classification, characteristics and application of hydrogels.

Classification		Sample	Trait	Application	Reference
Origin-based Classification	Natural hydrogel	Hyaluronic acid Sodium Alginate gelatin Agarose	Biocompatibility Environmental sensitivity Biodegradability	Tissuescaffold material Drug delivery	(Ishihara et al., 2002)
	Synthetic hydrogel	Acrylic acid and its derivatives polyvinyl alcohol (PVA) Polyethylene glycol and its copolymer	Easy for industrial manufacture chemical modification. Precise and controllable performance	Drugsustained release	(Czarnecka and Nowaczyk, 2020)
Crosslinking method	Chemical hydrogel	Acrylic acid series Acrylamide series	Free radical polymerization	Drug delivery	(Hussain et al., 2013)
	Physical hydrogel	Diphenylalanine	Physical interaction (hydrogen bonding, hydrophobic interaction, π-π stacking, metal ion coordination)	Drug delivery	(Lu et al., 2018)
Degradation performance	Degradable hydrogel	Most natural hydrogels	Biodegradability Biocompatibility	food additives Drug delivery	(Li et al., 2012)
	Nondegradable hydrogel	Most synthetic hydrogels	Easy for industrial production Chemical modification Precise and controllable performance	Soft tissue filling material Drug delivery	(Spiller et al., 2012)
Responsiveness	Environmental responsive hydrogel	Isopropyl acrylamide	Temperature-sensitive pH-sensitive Light-sensitive Electric-sensitive	Drug delivery	(Jiang et al., 2020)
	Nonenvironmentally responsive hydrogel	Agarose	Insensitive	Drug delivery	(Li et al., 2012)

**Figure 1.**
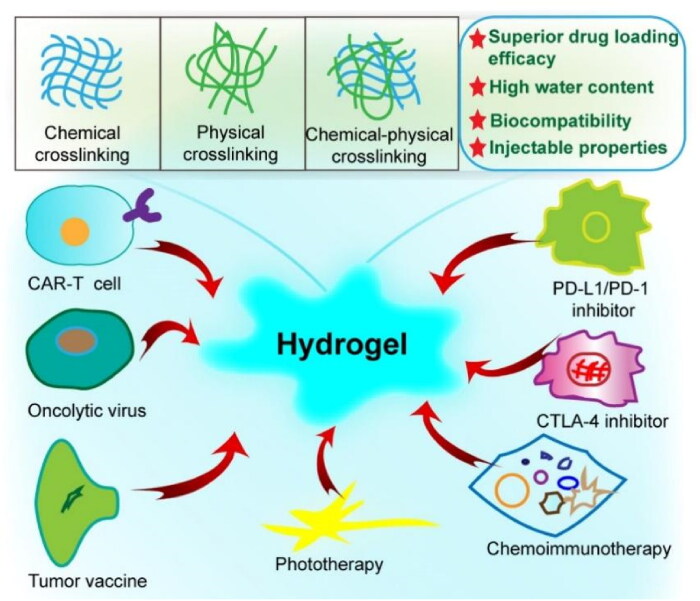
Illustration of the application of hydrogels in immunotherapy of gastrointestinal tumors. The crosslinking methods of hydrogels include chemical, physical and chemical-physical crosslinking. The review described the research progress on hydrogel delivery systems with immunotherapy methods such as checkpoint blockade therapy for gastrointestinal neoplasms.

Hydrogels are a very versatile class of materials with highly tunable physicochemical and biological properties that are suitable for tumor immunotherapy. Due to the distinct physical and chemical characteristics of hydrogels, the effect of tumor immunotherapy could be greatly augmented. First, hydrogels possess superior drug loading efficacy due to a large number of hydroxyl, carboxyl, and amino groups in the hydrogel, enabling it to provide a good platform for loading and releasing various drugs via direct drug loading or the incorporation of drug-loaded nanoparticles or microparticles (Choudhury et al., 2018; Hauptstein et al., 2020). For example, hydrogels could simultaneously incorporate antigens and immunogenic adjuvants, significantly increasing the immunogenicity of tumor vaccines, since these antigens could be enhanced by their fusion to highly immunogenic adjuvants (Li et al., 2022). Second, hydrogels are composed of hydrophilic polymer chains that absorb a large amount of water (Ahmed, 2015), and their water content can reach 90%, so they can provide a suitable physiological environment for drugs and have great advantages in the treatment of gastrointestinal tumors. Third, as a result of their high water content, hydrogels with excellent biocompatibility are able to imitate natural tissues (Salah et al., 2020). Moreover, it is possible to carry various types of cargoes through interactions between different sized interaction forces within the hydrogel network structure because of the availability of functional groups and adjustability of the structural hydrogel network (Qiu et al., 2020). Fourth, hydrogels possess injectable properties, which can give hydrogels the ability to be directly injected into the tumor site and increase the ability to directly stimulate tumor immunity. In addition, the injectable properties of hydrogels make them implantable in vivo through a rapid sol-gel phase transition or in situ chemical polymerization, which reduces surgical trauma and captures therapeutic molecules or cells at the target site in situ by simple syringe injection (Leach et al., 2019; Liu et al., 2020; Vigata et al., 2020). Finally, the ease of synthesis and low price of raw materials make hydrogels a very promising delivery carrier for tumor immunotherapy. Therefore, because of their unique properties, hydrogels have become a drug delivery system with a very high development prospect (Hori et al., 2008) (Figure 1).

## 3. Hydrogel-based immune checkpoint blockade therapy

T lymphocyte response intensity and quality are regulated by costimulatory factors and inhibitory signals in the cellular immune response of the body (Greenwald et al., 2005; Zou & Chen, 2008). Immunosuppressive signals are immune checkpoints that can affect the immune process and therefore regulate the steady state of the immune system. Overexpression of immune checkpoints inhibits T-cell activation and allows tumor cells to escape the immune system’s detection and destruction, leading to "immune escape". Therefore, immune checkpoint inhibitors (ICIs) were created, which prevented T cells from being inhibited by checkpoints, thereby killing tumor cells. A variety of tumors have been successfully treated by ICIs in recent years after they were developed and approved by the FDA (Kudo, 2019). Currently, immune checkpoint inhibitors can be divided into two categories: PD-1/PD-L1 inhibitors and CTLA-4 antibodies.

### 3.1. Hydrogel-based PD-1/PD-L1 therapy in gastrointestinal tumors

The immune system is capable of detecting and eliminating neoplasm cells under normal conditions, which inhibits the growth and development of cancer. T lymphocytes of the immune system can be weakened in the detection of cancer cells by malignant tumor cells through the PD-1/PD-L1 pathway and other pathways and elude immunosurveillance, thus achieving immune escape (Lussier et al., 2015; Wang et al., 2017). Consequently, by blocking tumor immune checkpoints, relieving tumor cell immunosuppression by cytotoxic T lymphocytes (CTLs), and restoring the function of the immune system of CTLs, the growth of tumors can be suppressed. Although immune checkpoint blockers have been clinically successful in the treatment of gastrointestinal tumors in recent years, only a small percentage of patients exhibit durable responses to monotherapy. The majority of the reasons are attributed to the strong immunosuppressed tumor microenvironment (TME) impeding the tumor response to immunotherapy (Cheever & Higano, 2011; Rosenberg et al., 2016). Using a hydrogel system to reprogram the immunosuppressive TME can effectively enhance the immune system. Chen et al. (2020) developed a shear-thinning nanofiber hydrogel based on betamethasone phosphate (Crusz & Balkwill, 2015) to reprogram the immunosuppressive TME and generate sustained PD-L1 receptor binding. As a nanofiber hydrogel based on steroids as anti-inflammatories, the inflammatory immunosuppressive TME can be reprogrammed through an increase in T lymphocyte infiltration as well as a decrease in immunosuppressive cells. Furthermore, the nanofiber hydrogel could also sustainably release PD-L1 under controlled conditions to strengthen T-cell immune responses against cancer cells, remarkably suppressing local neoplasm growth. Interestingly, it was also observed that this nanofiber hydrogel abscopically worked on distant neoplasms in a mouse model of colon tumors.

On the basis of hydrogel characteristics, an increasing number of environmentally responsive delivery formulations have been developed, of which temperature-sensitive hydrogels are the most common. As a thermoplastic hydrogel composite, Zhang et al. (2021) synthesized a polyethylene glycol-block-poly-L-leucine (PEGPLLeu) copolymer, which can be used for the synergistic treatment of rectal cancer with the continuous release of RCEFINI (REG) and BMS202 (PD-L1 inhibitor). At body temperature, polyethylene glycol polylactic acid microspheres are stable after sol-gel transformation. When combined with REG, BMS202 can withstand the immunosuppression caused by PD-L1 upregulation with no obvious side effects from REG. The orthotopic model of rectal cancer (CT26-Luc) showed that the double drug-loaded hydrogel was more effective than single drugs at reducing neoplasm growth. Additionally, amino acid-based hydrogels are capable of being degraded into neutral substances in vivo when exposed to various enzymes, which will not produce acidic metabolism at the neoplasm site to alter the pH value.

The current standard of care for peptides and antibody drugs for immunotherapy of gastrointestinal tumors is intravenous administration. Despite the fact that peptides have a higher degree of selectivity and fewer side effects than small molecules, they have poor half-lives and are difficult to administer orally. Based on this challenge, Li et al. (Zhu et al., 2021) designed an optimized polypeptide OPBP-1 (oral PD-L1 binding peptide 1) that was loaded upon an N, N,N-trimethyl chitosan (TMC) hydrogel for the treatment of colorectal cancer in mice. In the hydrogel delivery system, OPBP-1 exerts good blocking activity against PD-1/PD-L1 in vitro and antitumor activity in vivo. Moreover, oral administration of TMC hydrogel can greatly increase the penetration of polypeptide drugs into the gut, thereby increasing their oral bioavailability. These results indicate that anti-proteolytic PD-1/PD-L1 polypeptide inhibitors can be administered orally in the treatment of gastrointestinal tumors, which opens up new prospects for the development of oral drugs in tumor immunotherapy.

Based on clinical trials, PD-1/PD-L1 inhibitors have an effective rate of 80% against lymphoma and 60% against high-microsatellite instability (MSIH) neoplasms, and efficacy for other solid tumors ranges from 10% to 30% (Li et al., 2022). Therefore, it is very important to improve the efficiency of PD-1/PD-L1 in gastrointestinal tumors. The strategy of encapsulating ICIs into the hydrogel system can not only improve the immunotherapeutic response but also enhance the targeting effect. At the same time, various environmentally sensitive hydrogel carriers can directly deliver immunoactive ingredients and chemotherapy drugs to the tumor target at a low effective dose.

### 3.2. Hydrogel-based CTLA-4 therapy in gastrointestinal tumors

Similar to the costimulatory molecule CD28 expressed on T-cell surfaces, CTLA-4 (CD152) is an immune checkpoint transmembrane protein expressed on T cells. Antigen presentation is prevented by CTLA-4’s ability to compete with CD28 for B7 molecules (CD80 and CD86) on APCs, resulting in the disruption of T-cell activation and proliferation (Alegre et al., 2001). Blocking CTLA-4 receptors and their related immune pathways might release antitumor immunity to prevent tumor growth. Ultimately, it was the discovery of the role of anti-CTLA-4 therapy on antitumor immunity that led to ipilimumab being approved as the first and only anti-CTLA-4 immunotherapy by the FDA to date. Nevertheless, immune-related adverse events (irAEs) occurred in 60% of patients taking ipilimumab (Hodi et al., 2010). Thus, by developing controllable and targeted drug delivery gels, these limitations may be overcome, and anti-CTLA-4 therapy will be of greater benefit to more patient groups (Krysko et al., 2012; Pardoll, 2012; Sharma & Allison, 2015; Francis & Thomas, 2017; Overwijk, 2017; Chao et al., 2018; Grosser et al., 2019; Waldman et al., 2020). Kim et al. (Chung et al., 2020) used a poloxamer (P407) hydrogel to optimize the treatment of CTLA-4 for the first time. They prepared a hydrogel carrying CTLA-4 antibodies by mixing anti-CTLA-4 antibody with P407 in a physical manner, which was used to treat mouse CT-26 cells. According to the in vivo results, the P407 hydrogel showed good antitumor activity, which suppressed the growth of cancer cells and enhanced the survival rate of mice. In numerous clinical trials on various cancers, the CTLA-4 antibody ipilimumab has been combined with an anti-PD-1 antibody to demonstrate greater efficacy than single-agent treatment. By comparing the immune status of sensitive gastrointestinal tumors in a mouse model with resistance to ICIs, Mazoka et al. (Muraoka et al., 2019) described the mechanisms through which tumors resist T-cell-dependent immunotherapy. His study focuses on anti-PD-1 and anti-CTLA-4 immune checkpoint inhibitors used in the treatment of colon cancer CT-26. They constructed a new antigen delivery system by encapsulating a combination of three antibodies in cholesterol Pullulan (CHP) nanogels. Intravenous administration of this hydrogel induced antigen-presenting activity of TAMs intreated mice, overcame tumor immune resistance, and enhanced the accumulation of specific CD8^+^ T cells at the neoplasm site. The results demonstrate that a controlled hydrogel system not only maximizes the ability of CLTA-4 to attack tumors but also minimizes the need for repeated dosing, reducing irAEs and improving patient compliance. Moreover, it is a good strategy that the combined blockade of PD-1 and CTLA-4 by hydrogels is effective against several cancers. This finding creates new research avenues for optimizing tumor immunotherapy.

In light of the enormous amount of interest in drug development, thousands of clinical trials are underway that generate tens of thousands of data snapshots to help us understand how to utilize ICIs effectively. Ipilimumab, Nivolumab, and Pembrolizumab are currently the most commonly used ICIs in clinical practice. Checkmate-649, a Nivolumab based study, is the world’s first and largest phase III clinical trial of first-line immunotherapy for advanced gastric cancer (Janjigian et al., 2021). Nivolumab was approved as a first-line treatment for gastric cancer due to its positive overall results, but its clinical effectiveness still falls short of expectations. Currently, Infusion of intravenous drugs is still the most commonly used method of systemic drug delivery. How to build more drug delivery systems (such as hydrogel systems), improve the administration route of ICIs, and break through the current bottleneck of ICIs in the clinical treatment of gastrointestinal tumors is a very promising translational application.

## 4. Hydrogel-based adoptive T-cell therapy in gastrointestinal tumors

The adoption of cells or adoptive cell transfer (ACT) is a type of antitumor immunotherapy that is extremely attractive for its intelligence and patient-specific approach (Obajdin et al., 2020). ACT contains different steps to induce immune-mediated cancer clearance. First, circulating or tumor infiltrating lymphocytes are taken from patients. The cells were then selected, activated in vitro, genetically modified to express cancer-targeting receptors, and multiplied to a therapeutic level. After that, the reinjected cells were used to identify and eliminate cancer cells in the treated patient. ACT provides a variety of immune subtypes, including dendritic cell (DC), natural killer (NK), and T lymphocyte (T)-cell-based immunotherapies, and the clinical and preclinical stages of each are different (Rosenberg & Restifo, 2015; Patel et al., 2019; Waldman et al., 2020).

### 4.1. Hydrogel-based CAR-T-cell therapy in gastrointestinal tumors

Chimeric antigen receptor T cells (CAR-T) are a novel type of immune antitumor therapy that uses genetic engineering technology to modify T cells of patients, express CAR molecules on their surfaces, and then transfuse them into patients (Mohanty et al., 2019). Modified CAR-T cells can specifically identify and kill cancer cells and are currently one of the most advanced immune antitumor treatment methods (Mohanty et al., 2019).

**Figure 2.**
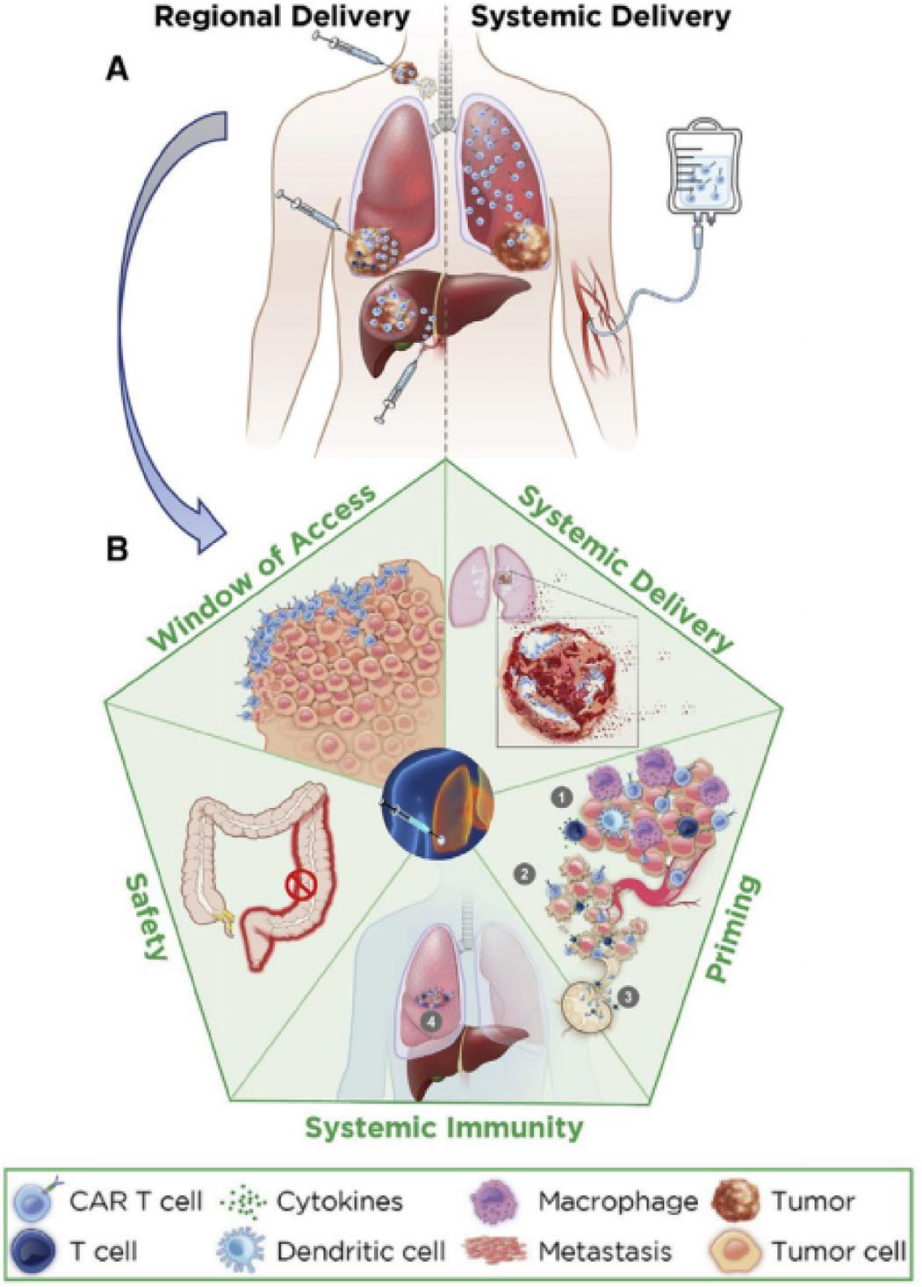
Regional versus systemic delivery of CAR-T cells. (A) Differences in CAR T cells administered regionally versus systemically. (B) The advantages of administering CAR-T cells regionally. Regional delivery enables CAR T cells to produce systemic immunity and more readily access metastatic areas, resulting in tumor lysis, and by inducing changes to the tumor microenvironment, it prepares the environment for systemic immunity. The strategy also accelerated the death of neoplastic cells, leading to an enhanced immune response and enhanced circulation of T cells in the tumor and entry into metastatic sites. In the case of regional delivery, long-term circulating T-cell immunity is caused by the local immune response, which eliminates systemic disease and prevents recurrence of neoplasms and minimizes toxic effects on the body. Reprinted from [Cancer Cell] Copyright 2022, with permission from Elsevier.

**Figure 3.**
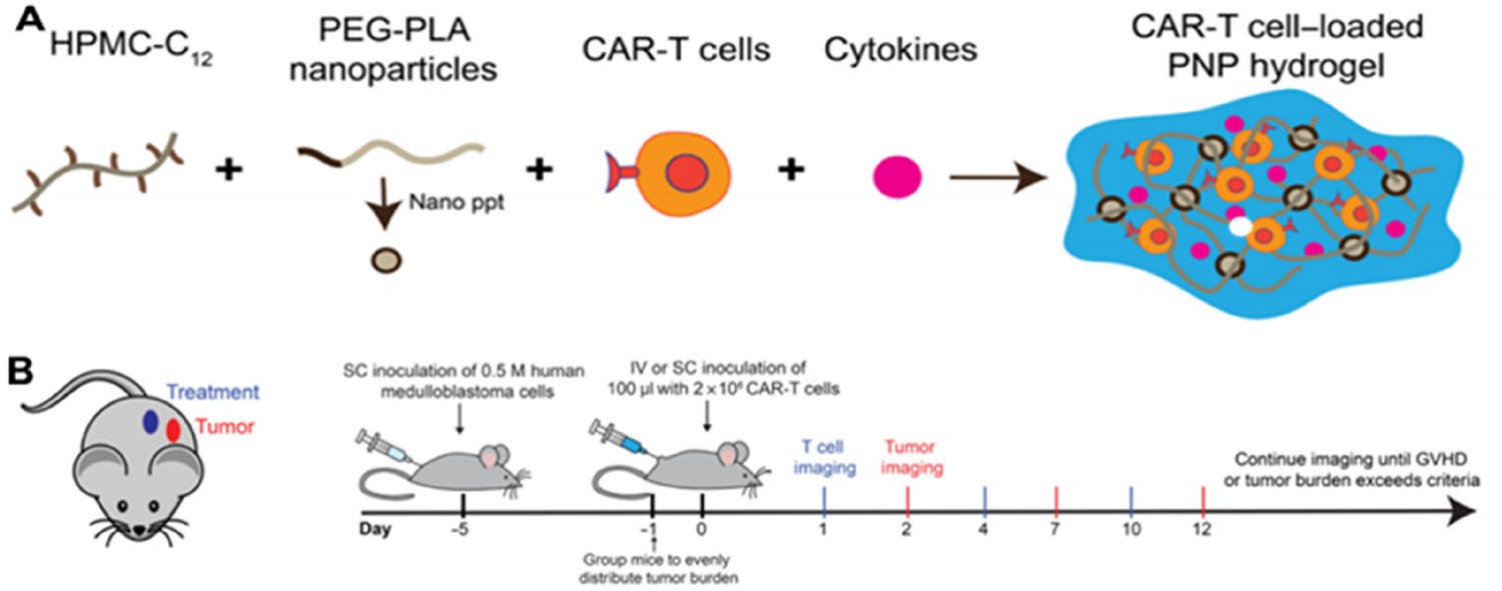
(A) Using dodecyl-modified hydroxypropyl methylcellulose and block-copolymer nanoparticles to form PNP hydrogels for coencapsulating CAR-T cells and stimulating cytokines. (B) Diagram showing the tumor’s location and the treatment injected. Experimental timeline for neoplasm experiments. Reprinted from [Science Advances] Copyright 2022, with permission from American Association for the Advancement of Science.

The success of CAR-T cells in treating hematological malignancies has raised the hope of using them in solid tumor therapy as well (Hoo et al., 2013). Despite this, the disappointing consequences of CAR-T therapy against gastrointestinal tumors are closely related to numerous obstacles (Rajendrakumar et al., 2018; Ternet & Kiel, 2021). After systemic administration of CAR-T cells, these cells display poor antitumor activity because they have difficulty identifying, infiltrating, and expanding within the immunosuppressive TME (Martinez & Moon, 2019). Moreover, T-cell dysfunction caused by anergy, ignorance, tolerance, or exhaustion is the most common obstacle. To overcome these barriers and create the circulatory immunity required for a lasting clinical answer, regional remedy has been used (Cherkassky et al., 2022). Regional delivery increases the intratumoral proliferation and penetration depth of CAR-T cells, allowing CAR-T cells to better enter the tumorigenesis locus, resulting in tumor lysis (Figure 2A and B). At the same time, to establish lasting, recyclable immunity to eradicate metastatic foci. Regional therapy is consistent with the local delivery of injectable hydrogels, so the application of hydrogels to deliver CAR-T cells to the region may be a promising direction of development. Grosskopf et al. (Grosskopf et al., 2022) designed a self-assembling and injectable biomaterial to deliver CAR-T cells based on polymer-nanoparticle (PNP) hydrogels (Appel et al., 2015; Stapleton et al., 2019; Grosskopf et al., 2020; 2021; Meis et al., 2021a; 2021b) (Figure 3). In addition to their high-scalability chemistry (Yu et al., 2016), these hydrogels can be formulated at mild temperatures and used to encapsulate CAR-T cells and cytokines without modification. The study showed that through their unique properties, these hydrogels can easily be injected directly into the body, creating a transient inflammatory niche in vivo that strengthens CAR-T-cell expansion and activation, leading to a profound improvement in efficacy in remedying solid tumors in mice. The study still demonstrated that the treatment was equally effective when administered proximally or distally to neoplasms, indicating that this strategy may be more suited to treat diseases such as metastases or tumors that are difficult to access through direct injection or catheter delivery.

Many CAR-T studies on solid tumors focus on how to make better cells but less on how to make CAR-T cells more effective and safer in vivo. As a result of regional administration, cytokine release syndrome occurred less frequently, proinflammatory cytokines were increased, and immunosuppressive cell quantities were decreased. Hence, regional administration of CAR-T cells via hydrogels can address an unmet need for effective treatment of solid tumors and enable effective therapy. Its modular and versatile design allows it to be applied to a wide variety of cell types and cytokine types, since the encapsulation and sustained delivery of cytokines do not require alteration of either. As a result of this key feature, injectable hydrogels can potentially be used as a basis for treating many solid tumors with CAR-T cells.

### 4.2. Hydrogel-based immune cell therapy in gastrointestinal tumors

In clinical trials of some patients with high-risk cancer, immunotherapy based on natural killer cells has been shown to be an effective means of protecting against malignant transformation (advanced melanoma, lymphoma, breast cancer and gastric cancer, etc.). Nevertheless, only a few patients with specific neoplasms have responded favorably to immunotherapy to date (Lim & June, 2017; Liu et al., 2020). In vitro cancer models using two-dimensional (2 D) culture systems or animal models have contributed to the development of anticancer treatments for many years (Shoemaker, 2006; Siolas & Hannon, 2013). However, these models have some limitations in that they do not contain the tumor microenvironment (TME) components and do not take into account species-dependent variations. In immune surveillance, cytotoxic lymphocytes, including NK cells, are significant. A constant circulation of these cells in the blood detects and destroys cancerous cells (Waldhauer & Steinle, 2008). Hence, it is urgent to develop in vitro models that capture the intricate interactions between neoplasms and their surrounding microenvironment in the tumor vasculature. Based on hydrogels, Song et al. (2021) developed a 3 D extracorporeal tumor model that incorporates colorectal cancer (CRC) cells surrounding perfusable vascular networks for testing immune-cell-mediated cytotoxicity against neoplasm cells. The biomaterials consist of 28 microwells that can be used to create identical vascularized cancer models. Hydrogel patterns can be robustly patterned for high-throughput experiments in 3 D cultures with this technique. This study demonstrated the capability of the biomaterial by introducing NK cells into a vascularized tumor network that was made of hydrogel and monitoring their activity through live-cell imaging. Comparative tests were performed on six kinds of CRC cell lines to assess extravasation, migration, and cytotoxic activity.

In the immunosuppressive microenvironment, many immune cells infiltrate, including regulatory T (T-reg) cells, MDSCs and tumor-associated macrophages (TAMs), to restrain antitumor immune responses (Rosenberg, 2001; Binnewies et al., 2018; Waldman et al., 2020). Rebuilding the tumor immune microenvironment by overturning the repressive effects of immune cells and reducing the presence of immunosuppressive cells would therefore be beneficial in overcoming immunosuppression resistance.

**Figure 4.**
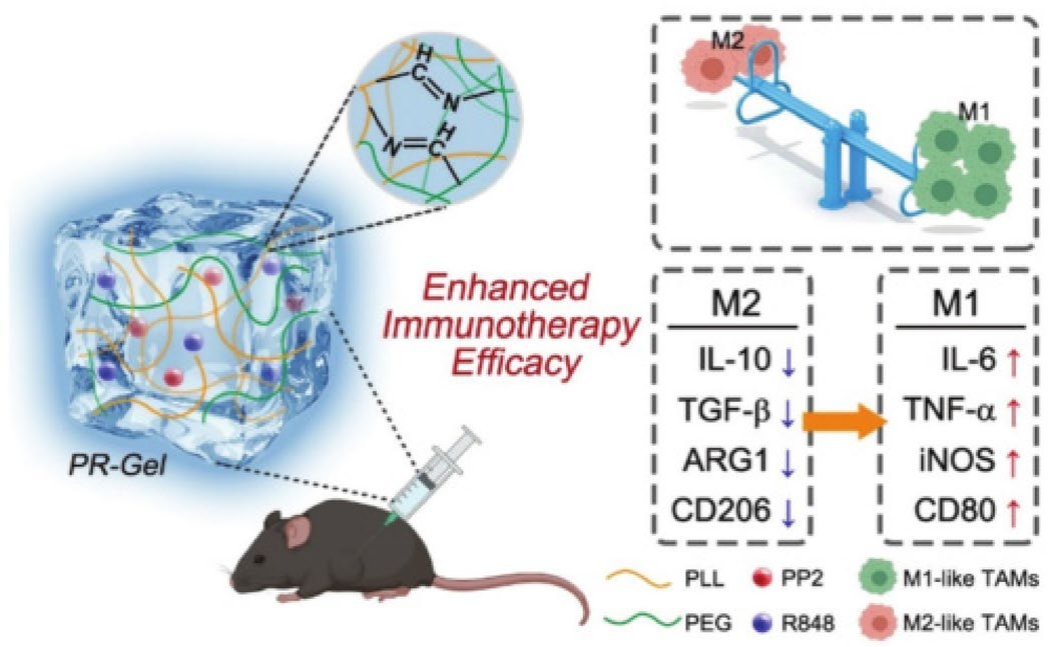
The PR-Gel schematic illustrates how to repolarize. TAMs and enhances gastric tumor immunotherapy. The study tested RAW264.7 cells for phenotypic transition and cytokine secretion following PR-Gel treatment to determine whether PR-Gel repolarizes M2-to-M1 TAMs. In a xenograft mouse model of gastric cancer, PR-Gel was evaluated for its ability to enhance the antitumor immune response and effectiveness by a single subcutaneous injection. Reprinted from [Biomaterials Science] Copyright 2021, with permission from the Royal Society of Chemistry.

M2-like TAMs could be critically important for reprogramming the immunosuppressive TME toward proimmunogenicity for the feature of immune cells and for improving immunotherapy (Xia et al., 2020; Raskov et al., 2021). Thus, it would be an attractive strategy to reprogram M2-like TAMs into M1-like macrophages to restore macrophage antitumor properties. The hydrogels can be placed at the locus of a neoplasm and act as cargo depots to deliver therapeutic agents in a controlled manner (Norouzi et al., 2016; Xiao et al., 2021). Shear thinning properties are possessed by cross-linked hydrogels with dynamic covalent bonds, and they can be injected directly into neoplasms without invasive surgery or implantation (Qian et al., 2019; Kumar & Bajaj, 2020). As part of gastric tumor immunotherapy, Yang et al. (2021) designed an injectable shear-thinning hydrogel containing polyphyllin II (PP2) and resiquimod (R848) (referred to as PR-Gel) (Figure 4). An improved drug absorption rate at the tumor tissue could be achieved with PR-gel, resulting in long-term drug remission. PR-Gel in particular was able to induce a shift from M2-like macrophages to M1-like macrophages, leading to strengthened tumor immunity and restrained neoplasm growth. Although this treatment strategy for tumors has many advantages, repolarizing TAMs is still not good enough to eradicate gastrointestinal tumor cells. Therefore, the combination of PR-GEL with other therapeutic drugs, such as anti-PD-1/PD-L1, may be a viable approach to entice neoplasm recession through congenerous effects in the future.

## 5. Hydrogel-based tumor vaccines in gastrointestinal tumors

The role of tumor vaccines in the early phases of the immune response period has drawn widespread attention because they use neoplasm-specific antigens to stimulate T-cell-mediated immune responses. Tumor vaccines are generally formulated using tumor-associated antigens (TAAs) and adjuvants, which reactivate the immune system’s response to tumor cells and produce persistent antitumor efficacy. Tumor vaccines have shown inspiring potential in tumor immunotherapy by enhancing antitumor immunity that lasts for a long time. However, there are still a number of limitations for cancer vaccines, such as neoantigen discernment, low immunogenicity, and weak immune reaction (Kuai et al., 2017; Hu et al., 2018). To date, cancer vaccines have rarely achieved an effective response in most patients with gastrointestinal tumors. It is, thereby, necessary to use advanced technologies to ameliorate the mild and momentary immune response for gastrointestinal tumor patients. In addition to being used as a chemotherapeutic sustained-release system, hydrogels can also be used as cancer vaccine carriers (Adu-Berchie & Mooney, 2020). With a hydrogel delivery system, the antigen is protected from degradation and delivered to dendritic cells (DCs) and T lymphocytes in a targeted way, as opposed to direct injection, and produces cross-presentation of antigens. Thus, cytotoxic T lymphocyte production is increased, and antitumor immunity is enhanced (Sehgal et al., 2014). Yang et al. (2021) designed a combined immunotherapy strategy utilizing PLEL hydrogels capable of responding to temperature changes. They mixed the cyclo-phosphamide and poly (L-lactide)-poly (ethylene glycol)-poly (L-lactide) hydrogel (PLEL) and injected it intratumorally. The cancer cells were killed by the sustained release of cyclophosphamide at the tumor site, and the dead cancer cells released TAAs and immunostimulatory danger signals, which were identified and absorbed by dendritic cells (DCs), thereby exerting antitumor immunity. After a while the infection was coloaded into the PLEL hydrogel along with the adjuvant cytosine-phosphate-guanine oligonucleotide (CpG ODN) and injected subcutaneously into bilateral groins, the hydrogel neoplasm vaccine forms. Continuous release of tumor antigens and adjuvants occurs when the gel forms at body temperature. It facilitated the migration of DCs when they ingested and recognized tumor antigens to the hydrogel vaccine, so these “modified” DCs can become more vivacious and mature, which can boost the antitumor response (Worbs et al., 2017). Furthermore, the systemic antitumor immunity mediated by the local delivery of this PLEL hydrogel strategy suppressed the growth of tumors in distant parts of the body. By combining these two treatment strategies, the antitumor efficacy of the neoplasm lysate-based neoplasm vaccine is enhanced, as well as reducing the toxicity and side effects of conventional chemotherapy; thus, tumor immunotherapy can be aided by this approach, which provides a new concept for combining chemotherapy with cancer vaccines of gastrointestinal tumors.

Tumor vaccines represent a promising and cost-effective antitumor strategy. However, there are many obstacles that restrict the clinical efficacy of tumor vaccines, including the lack of optimized delivery systems for generating enduring and intense immune responses against neoplasms. It is a new concept for combining chemotherapy drugs with tumor vaccines by a hydrogel system that the antitumor impact of the neoplasm lysate-based tumor vaccine is enhanced, and the toxicity and side effects of conventional chemotherapy are obviously reduced.

## 6. Hydrogel-based oncolytic viruses in gastrointestinal tumors

Oncolytic viruses (OVs) are emerging as promising and potential anticancer therapeutic agents (Deng et al., 2021). OVs are capable not only of eliminating cancer cells directly by replicating selectively within the cell but also of promoting the immune response against tumors (Marelli et al., 2018). Through the use of a cell defect inherent in tumorigenesis, oncolytic viruses selectively infect, replicate, and destroy cancer cells (Prestwich et al., 2009; Russell et al., 2012; Kaufman et al., 2015). The first OV talimogene-laherparepvec (T-VEC) immunotherapy for melanoma was approved by the FDA in October 2015. Various related OVs are being developed for different types of tumors and are in different phases of preclinical and clinical trials. It is unfortunate that there is a limited amount of bioavailable OVs because the systemic injection of OVs can stimulate an intense immune response, which can be neutralized by antibodies in the bloodstream. Consequently, it reduces circulation time and is eventually excreted from the body quickly, resulting in low therapeutic efficacy. Recently, several studies (Jenner et al., 2020; Jung et al., 2019) have demonstrated the potential of hydrogels for the effective local and standing delivery of OVs because they are biocompatible, have high water retention (for maximum absorption), are mechanically similar to tissues, circulate in the bloodstream for a long period of time, have a targetable surface that can be modified, and have the capability to be dispersed for systemic administration (Chyzy et al., 2020). Therefore, Deng et al. (Deng et al., 2021) developed a hyaluronic acid-based, redox-responsive nano hydrogel to deliver OVs, aiming to protect OVs after systemic administration. ECHO-7 (RNA virus) was chosen as the normative oncolytic virus and then loaded into a nanohydrogel. The redox response characteristic of this hydrogel drug delivery system resulted in its degradation under conditions of reduction, including intracellular or tumor environments, leading to apoptosis in HT-29 colon cancer cells. The results indicate that the nanohydrogel delivery system can serve as an anti-OV protective cargo and a carrier to achieve systemic drug delivery and locus-specific targeting of oncolytic immune virus therapy. A novel strategy of systemic clinical transformation treatment for gastrointestinal tumors may be achieved through the combination of hydrogel technology with oncolytic immunotherapy.

In addition to systemic drug delivery, diverse effector cells and an oncolytic adenovirus expressing antitumor cytokines can be injected into tumors to exert an effective immunotherapy influence by oncolysis and transforming the tumor microenvironment. However, certain limitations were associated with this combination therapy. A large number of nontarget tissues can be infiltrated by effector cells and oncolytic viruses when the combination therapy is used at high concentrations. Moreover, because of the immunogenic nature of both treatments and their shorter bioactivity, multiple administrations were required to achieve a satisfactory curative effect. To overcome these barriers, Du et al. (Du et al., 2022) encapsulated a gelatin-based hydrogel capable of codelivery of an oncolytic adenovirus containing IL12, IL15 and CIK cells to enhance and prolong the antitumor effects of combined treatments after a single intratumoral injection in a colon cancer model. With an injectable, biodegradable hydrogel encapsulating high-dose oncolytic adenovirus, possible dispersion of the virus and CIK cells to the liver and nontarget tissues is reduced, and an effective antitumor immune response is continuously induced with only one single dosing ratio.

In summary, the strategy of combining a hydrogel system with oncolytic immunotherapy is expected to provide a new idea for both systemic and localized clinical transformation treatment in gastrointestinal tumors.

## 7. Hydrogel-based chemoimmunotherapy in gastrointestinal tumors

Chemoimmunotherapy (CIT) is a potentially powerful therapy that combines both conventional chemotherapy and present immunotherapy methods to suppress tumor growth, metastasis, and reappearance (Emens, 2010; Gandhi et al., 2018). In this new approach, chemotherapy kills cancer cells and produces cross-presented tumor antigens, thereby making cancer cells themselves a source of tumor antigens. Afterwards, the concurrent or successive injection of immunotherapeutic agents causes the tumor antigens and immune stimulants to interact to generate a strong antitumor immune response (Lake & Robinson, 2005). Furthermore, immunotherapy can overcome the drawbacks of chemotherapy resulting from poor specificity and high drug tolerance, as well as improve the sensitivity of cancer cells to chemotherapy (Ewens et al., 2006; Heo et al., 2015). Therefore, by combining these medications, therapeutic effectiveness could be increased through synergistic effects. As a new drug delivery strategy, injectable hydrogels offer a universal and effective platform for concurrent or continuous regional joint delivery of chemotherapeutic cargoes and immunomodulators.

**Figure 5.**
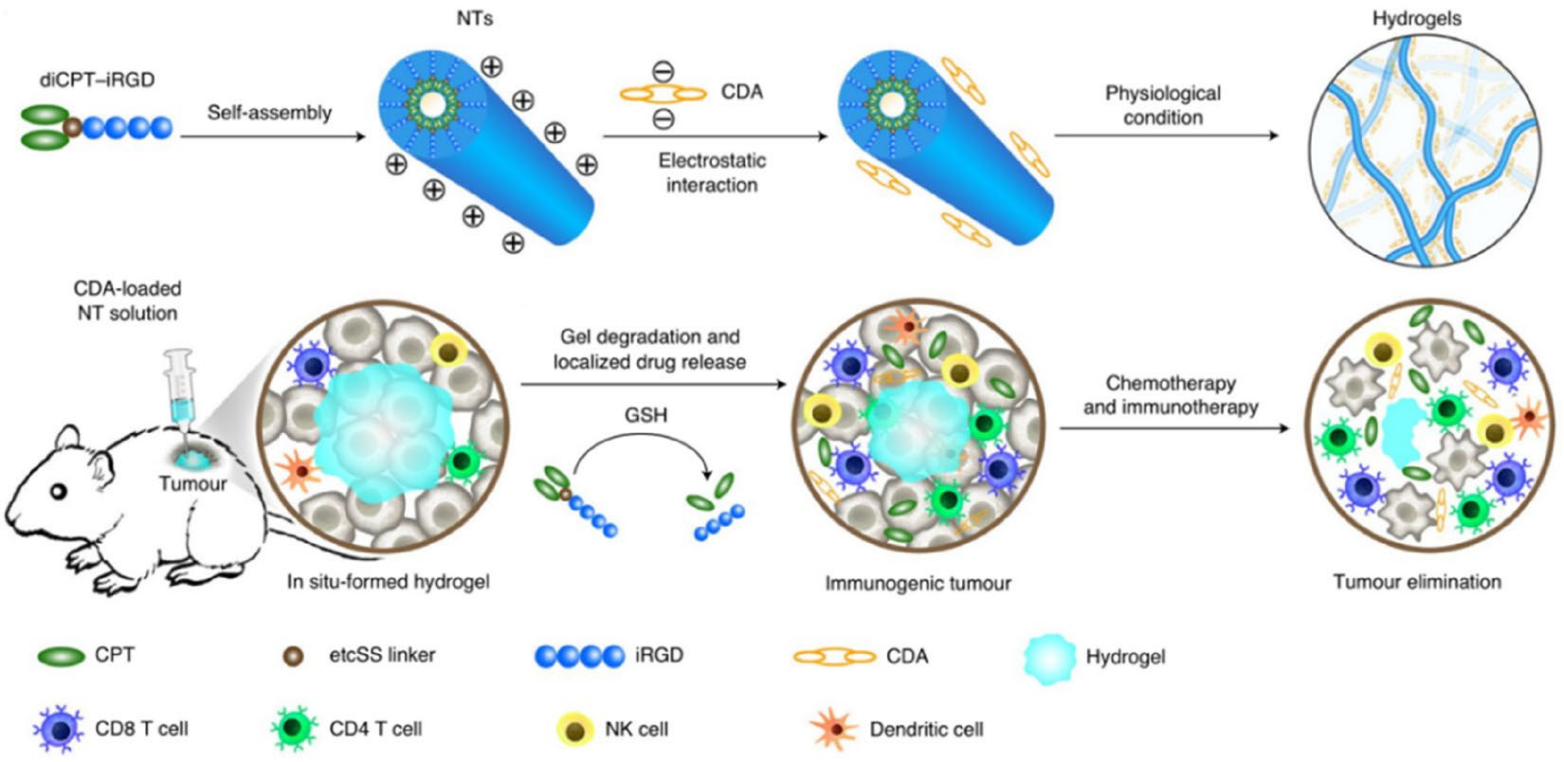
Schematics of localized CPT and CDA delivery using a nanotube hydrogel for TME regulation and chemoimmunotherapy. The peptide iRGD was chemically conjugated with the hydrophobic anticancer drug CPT to form diCPT–iRGD, which self-assembles as a peptide–drug conjugate. Supramolecular nanotubes (NTs) were formed spontaneously in aqueous solution by the designed drug amphiphile. The negatively charged STING agonist (c-di-AMP (CDA)) can condense on the surface of these positively charged NTs through electrostatic complexation. After injection into the tumor site, the CDA–NT solution can immediately form a hydrogel, functioning as a local reservoir for extended localized release of CDA and CPT to awaken both the innate and adaptive immune systems. Reprinted from [nature biomedical engineering] Copyright 2020, with permission from Springer Nature.

Wang et al. (2020) developed a drug-bearing supramolecular hydrogel system to intratumorally (i.t.) deliver CDNs against malignant solid tumors to reach cancer chemoimmunotherapy (Figure 5). His strategy was to chemically conjugate the hydrophilic peptide moiety iRGD (a tumor-penetrating peptide that can bind to neuropilin-1 and stimulate tumor tissue penetration) (Sugahara et al., 2009) to the hydrophobic anticancer cargo CPT. After administration into the tumor locus, both CDA and CPT can be released at a localized level through the formation of a hydrogel, providing an extended localized release of both agents, thereby triggering both innate and adaptive immune responses. CDA and CPT can be delivered to tumor sites with ease by this in situ-formed CDA-NT hydrogel; thus, NK cells, DCs, and T cells are activated, and the overall survival of mice with colon cancer is increased. This improvement may be due to the longer release of STING agonists. Moreover, the invasion of these immune cells forms an immune-derived niche, which is critical to the development of antitumor efficacy. Furthermore, local administration of the hydrogel induces long-term immune memory and systemic immune surveillance, thereby preventing tumor recurrence and metastasis. These results show that this localized chemoimmunotherapy hydrogel offers the possibility to augment the antitumor immune response and to sensitize tumors to immunotherapies in an effective and safe mode.

Currently, for late-stage cancers with distant metastases, chemoimmunotherapy with reduced systemic toxicity and strong efficacy is essential. Several "cocktail" formulations have been reported that combine chemotherapeutics inducing immunogenic cell death (ICD) with immune adjuvants and alginate to induce localized chemoimmunization therapy (Chao et al., 2020). The ICI antibody can either be included in this cocktail for local administration or injected intravenously. The gelation of ALG in place after injection into solid tumors would induce local retention and sustained release of therapeutics, thereby reducing the systemic toxicity of the treatment. With the help of immune adjuvants, chemotherapy-induced ICD triggers an immune response specific for tumors, which is magnified by ICIs to produce activated systemic immune responses against cancer in devastating regional tumors, eliminating metastases and restraining cancer reappearance. The tumor-localized cocktail chemoimmunotherapy strategy that integrates clinically common agents holds great promise for clinical translation.

The results of clinical and basic research support the rationality of the combination of chemotherapy and immunotherapy. In particular, the use of a hydrogel system, giving full play to the characteristics of hydrogels and integrating immunotherapy agents with chemotherapeutic cargoes, has immense potential as a long-range maintenance remedy for cancer to maximize the congenerous antitumous effect.

## 8. Hydrogel-based phototherapy in gastrointestinal tumors

Photothermal therapy (PTT) and photodynamic therapy (PDT), as two main strategies of phototherapy, have been widely investigated for their efficient local tumor clearance in a spatiotemporally controllable manner and have received much attention in the therapeutic application of gastrointestinal tumors. The method of photothermal therapy involves irradiating materials such as near-infrared (NIR) light that are highly photothermal convertible with light to kill cancer cells without invasive procedures, which causes a local thermal effect at the tumor site (Fan et al., 2016; Zhu et al., 2017). Because NIR radiation induces cell death by apoptosis and necrosis by irradiating absorbance-delivered cancer cells, it can significantly reduce undesirable side effects, such as damage to healthy tissues (Dong et al., 2019). A novel injectable hydrogel with photothermal-sensitive properties was developed by Zheng et al. (2019) for safe and highly effective colon cancer hyperthermia and chemotherapy in vivo. When chitosan solution was injected into the tumor at room temperature, it automatically gels upon warming to body temperature when present in conjunction with β-glycerophosphate. Combining localized tumor photothermal treatment and chemotherapy was achieved by dissolving bi2s3-pEG (MBP) nanosheets in a hydrogel and incorporating the active cargo doxorubicin (DOX), and the gel system can encapsulate DOX and MBP nanosheets, preventing them from entering the bloodstream and potentially injuring normal tissue and cells.

Unlike PTT, photodynamic therapy kills tumors by producing cytotoxic ROS in cooperation with light irradiation, photosensitizer and tissue oxygen (Crous & Abrahamse, 2013). Notably, it has been proven that TAAs produced by PTT or PDT can improve the antitumor immune response. However, the antitumor immune response caused by PDT is usually moderate, and the therapeutic effect of PDT on solid tumors, especially gastrointestinal tumors, is still weak. In photoimmunotherapy, nanomaterials have already been widely used due to their carrier advantage, imaging capabilities, and therapeutic properties. Despite their high surface reactivity, nanomaterials "as prepared" suffer from poor biocompatibility and short plasma half-life when used in vivo. By encapsulating these nanomaterials in hydrogels, this challenge is well solved. Therefore, the regional combination of PTT/PDT and immunotherapy based on a hydrogel delivery system has been studied for tumor treatment (Castano et al., 2006; Chen et al., 2016).

**Figure 6.**
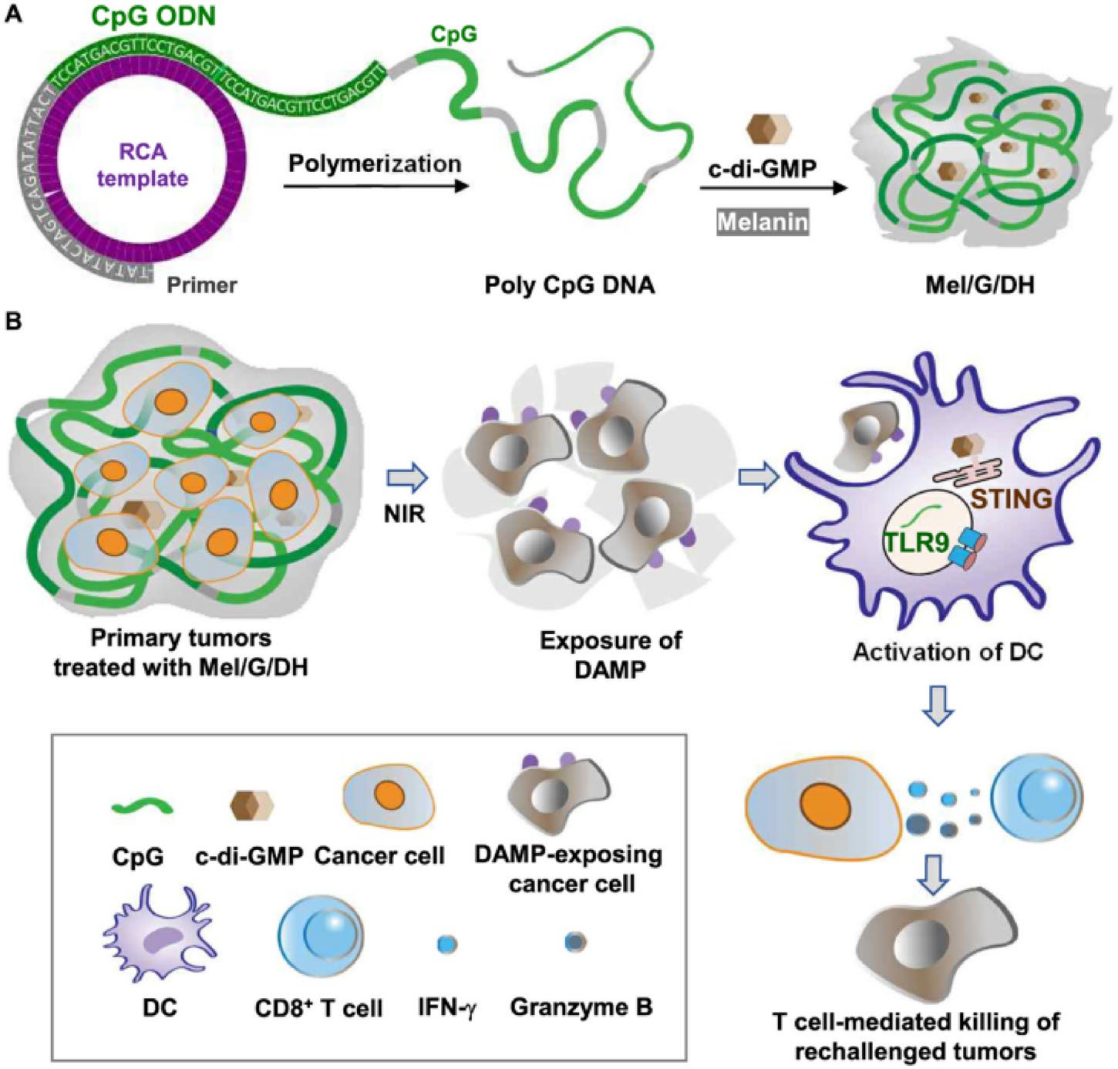
Proposed action mechanism of melanin-loaded DNA adjuvant hydrogel. (A) Schematic illustration of the Mel/G/DH hydrogel preparation process. (B) Mel/G/DH hydrogel was injected intratumorally. Upon near infrared (NIR) irradiation, DAMP signals (e.g., calreticulin) were exposed on the cancer cell surface, and dying cancer cells were phagocytosed by DCs. The dual adjuvants c-di-GMP and CpG were released from the hydrogel to act as potent immunostimulators for DC maturation and antigen presentation in tumor-draining lymph nodes, leading to the differentiation of immature T cells into CD8+ T cells. Subsequently, CD8+ T cells infiltrated into secondarily challenged tumors and prevented tumor recurrence. Reprinted from [J Control Release] Copyright 2020 with permission from Elsevier.

Wu et al. (2021) designed a biodegradable and immunostimulable DNA hydrogel that was used to deliver dinucleotide adjuvant and photoresponse components to treat colorectal cancer synergistically through photothermal and immunotherapy (Figure 6). They synthesized a DNA CpG hydrogel (DH, produced by rolling circle expansion), loaded it with bis-(3′-5′)-cyclic dimeric guanosine monophosphate (G/DH), and coated the formula with melanin (Mel/G/DH). It was proven that the photothermal hydrogel based on rolling circle amplification (RCA) showed an enhanced immunotherapy effect through photothermal therapy-mediated in situ vaccination and double adjuvant to DC. Therefore, the combination of immunotherapy and phototherapy in the treatment of primary gastrointestinal tumors through a hydrogel system can not only cause tumor cells to undergo photothermal ablation but also prevent distant tumor recurrence through immunotherapy.

In conclusion, hydrogels have natural advantages in simulating the cell microenvironment due to their hydrophilicity. Most polymers can be modified with photoresponsive groups, and then a variety of light reactions occur under light irradiation. Therefore, many materials can be prepared by adding photoresponsive groups that can play an important role in gastrointestinal tumor immunotherapy.

## 9. Conclusion and prospective

Therapeutic advances in tumor immunotherapy have rapidly developed in the past several years, reflecting the importance of the interaction between the human immune system and malignant tumorigenesis. Despite the successful application of tumor immunotherapy across a broad range of human cancers, only a minority of patients with gastrointestinal tumors received durable overall survival from these therapies. Hydrogels have emerged as a new research direction in recent years for antitumor drug delivery because of their unique characteristics, providing a prospecting treatment strategy for gastrointestinal tumors. Researchers are also designing matrix materials to endow gel with different chemical and physical properties so it can adapt to different environments following surgery, and by using encapsulated therapeutic agents, radiotherapy/chemotherapy, photothermal therapy, immunotherapy and other strategies to combat tumors are realized. In particular, hydrogels combining immune checkpoint inhibitors, adoptive cell therapy, and tumor vaccines have the potential to not only activate the immune system and prevent metastasis from spreading throughout the body but also prevent local recurrence, which may be an important trend of future application (Ishikawa et al., 2017). This review emphasizes recent progress in hydrogel systems for immunotherapy of gastrointestinal tumors.

In view of the low response rate and potential irAEs of ICIs such as PD-1/PD-L1 in the treatment of gastrointestinal tumors. The hydrogel system can boost the host’s immune system against tumors and strengthen the effect of immunotherapy. Diverse immunomodulatory agent combinations have been established, as well as local delivery of dual or multiple immunomodulatory cargoes. In addition, in different tumor models, immunotherapeutic cargo combinations in hydrogel systems resulted in significantly heightened antitumor immune responses, especially ICI antibodies, with conventional antitumor approaches.

Different from previous research ideas, the unique nature of hydrogels makes it easy to administer drugs by direct injection and creates a short-term inflammatory niche in vivo to enhance the expansion and activation of CAR-T cells and significantly improve the efficacy of treating solid tumors in mice. These hydrogels utilize highly scalable chemicals that can be modulated under moderate circumstances to easily encapsulate CAR-T cells and cytokines without modifying the cargo.

Using the characteristics of hydrogels can improve the mild and transient immune response of the host. We can also design hydrogels with different environmental sensitivities and improve immunogenicity by combining the effects of immunogenic cell death induced by chemotherapy drugs. Therefore, the hydrogel system can be used not only as a slow-release system for chemotherapy but also as a carrier for cancer vaccines. It can not only reduce the toxicity and side effects of conventional chemotherapy but also enhance the antitumor effect of tumor vaccines.

Despite the immense prospect of hydrogel-based delivery systems for antitumor immunotherapy, there are still some shortcomings in the development of a hydrogel drug delivery system to treat gastrointestinal tumors. First, most hydrogel drug delivery systems use in vivo testing on subcutaneous ectopic tumors, and further experiments in in situ tumor models are needed to investigate efficacy in vivo. Second, there are no long-term safety reports on the hydrogel system in vivo. Finally, hydrogel drug delivery systems are complex, and they are difficult to manufacture in large quantities or control their quality, so further optimization is needed.
